# 2-(4-Chloro­phen­yl)-*N*-(1,3-thia­zol-2-yl)acetamide

**DOI:** 10.1107/S1600536812034629

**Published:** 2012-08-11

**Authors:** Hoong-Kun Fun, Ching Kheng Quah, Prakash S. Nayak, B. Narayana, B. K. Sarojini

**Affiliations:** a X-ray Crystallography Unit, School of Physics, Universiti Sains Malaysia, 11800 USM, Penang, Malaysia; b Department of Studies in Chemistry, Mangalore University, Mangalagangotri 574 199, India; c Department of Chemistry, P. A. College of Engineering, Nadupadavu, Mangalore 574 153, India

## Abstract

In the title compound, C_11_H_9_ClN_2_OS, the thia­zole ring is nearly planar (r.m.s. deviation = 0.003 Å) and forms a dihedral angle of 64.18 (7)° with the bezene ring. In the crystal, inversion dimers linked by pairs of N—H⋯N_t_ (t = thia­zole) hydrogen bonds generate *R*_2_^2^(8) loops.

## Related literature

For general background to the title compound and for related structures, see: Fun *et al.* (2011*a* ▶,*b* ▶, 2012*a* ▶,*b* ▶). For hydrogen-bond motifs, see: Bernstein *et al.* (1995 ▶).
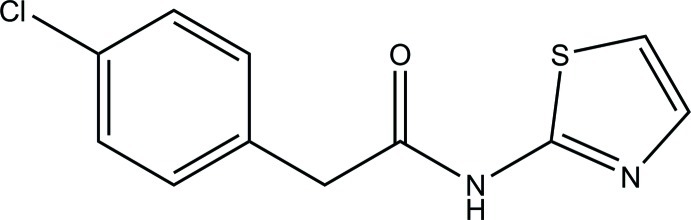


## Experimental

### 

#### Crystal data

C_11_H_9_ClN_2_OS
*M* *_r_* = 252.71
Monoclinic, 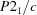

*a* = 13.9169 (13) Å
*b* = 5.5188 (5) Å
*c* = 15.1836 (14) Å
β = 100.311 (2)°
*V* = 1147.34 (18) Å^3^
*Z* = 4
Mo *K*α radiation
μ = 0.49 mm^−1^
*T* = 296 K
0.35 × 0.29 × 0.18 mm

#### Data collection

Bruker SMART APEXII DUO CCD diffractometer
Absorption correction: multi-scan (*SADABS*; Bruker, 2009 ▶) *T*_min_ = 0.847, *T*_max_ = 0.918
11463 measured reflections
3421 independent reflections
2769 reflections with *I* > 2σ(*I*)
*R*_int_ = 0.020

#### Refinement

*R*[*F* ^2^ > 2σ(*F* ^2^)] = 0.036
*wR*(*F* ^2^) = 0.116
*S* = 1.02
3421 reflections
149 parameters
H atoms treated by a mixture of independent and constrained refinement
Δρ_max_ = 0.29 e Å^−3^
Δρ_min_ = −0.29 e Å^−3^

### 

Data collection: *APEX2* (Bruker, 2009 ▶); cell refinement: *SAINT* (Bruker, 2009 ▶); data reduction: *SAINT*; program(s) used to solve structure: *SHELXTL* (Sheldrick, 2008 ▶); program(s) used to refine structure: *SHELXTL*; molecular graphics: *SHELXTL*; software used to prepare material for publication: *SHELXTL* and *PLATON* (Spek, 2009 ▶).

## Supplementary Material

Crystal structure: contains datablock(s) global, I. DOI: 10.1107/S1600536812034629/hb6927sup1.cif

Structure factors: contains datablock(s) I. DOI: 10.1107/S1600536812034629/hb6927Isup2.hkl

Supplementary material file. DOI: 10.1107/S1600536812034629/hb6927Isup3.cml

Additional supplementary materials:  crystallographic information; 3D view; checkCIF report

## Figures and Tables

**Table 1:** Hydrogen-bond geometry (Å, °)

*D*—H⋯*A*	*D*—H	H⋯*A*	*D*⋯*A*	*D*—H⋯*A*
N2—H1*N*2⋯N1^i^	0.866 (18)	2.096 (18)	2.9606 (16)	176.2 (17)

Symmetry code: (i) 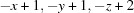.

**Fig. 1.**
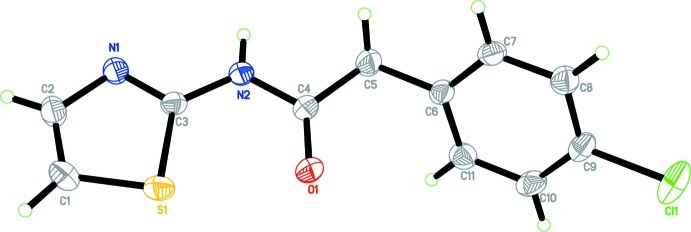
The molecular structure of the title compound showing 30% probability displacement ellipsoids for non-H atoms.

**Fig. 2.**
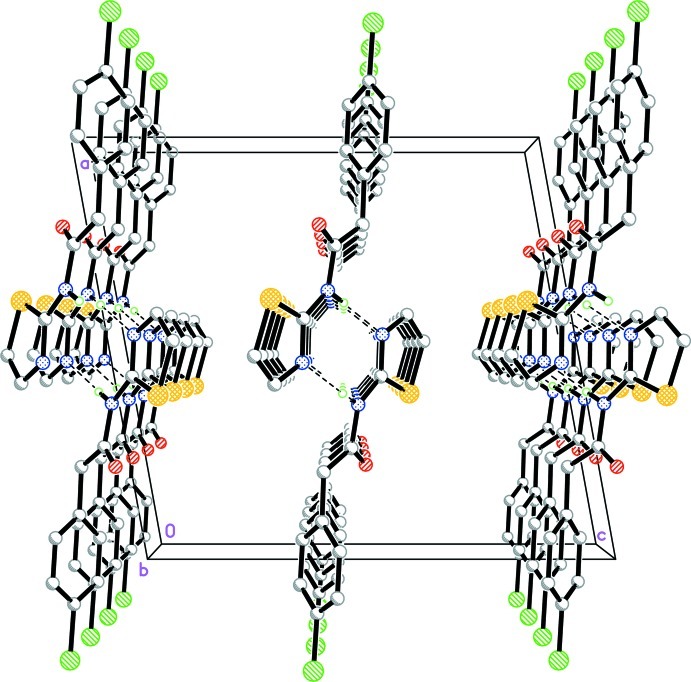
The crystal structure of the title compound, viewed along the b axis. H atoms not involved in hydrogen bonds (dashed lines) have been omitted for clarity.

## supplementary crystallographic information

### Comment

In continuation of our work on synthesis of amides (Fun *et al.*, 2011*a*, 2011*b*, 2012*a*, 2012*b*), we report herein the crystal structure of the title compound.

In the title molecule (Fig. 1), the thiazol-2-yl ring (S1/N1/C1-C3) is nearly planar (r.m.s. deviation = 0.003 Å) and it forms a dihedral angle of 64.18 (7)° with the bezene ring (C6-C11). Bond lengths and angles are within normal ranges and are comparable to related structures (Fun *et al.*, 2011*a*, 2011*b*, 2012*a*, 2012*b*).

In the crystal structure, Fig. 2, molecules are linked into an inversion dimer by pairs of N2–H1N2···N1 hydrogen bonds (Table 1), generating R_2_^2^(8) ring motifs (Bernstein *et al.*, 1995).

### Experimental

4-Chlorophenylacetic acid (0.170 g, 1 mmol), 2-aminothiazole (0.1 g, 1 mmol) and 1-ethyl-3-(3-dimethylaminopropyl)-carbodiimide hydrochloride (1.0 g, 0.01 mol) were dissolved in dichloromethane (20 ml). The mixture was stirred in presence of triethylamine at 273 K for about 3 h. The contents were poured into 100 ml of ice-cold aqueous hydrochloric acid with stirring. The resulting solution was extracted thrice with dichloromethane. The organic layer was washed with saturated NaHCO_3_ solution and brine solution, dried and concentrated under reduced pressure to give the title compound (I). Orange blocks were grown from an acetone and toluene (1:1) solvent mixture by the slow evaporation method (*m.p.*: 441K).

### Refinement

Atom H1N2 was located in a difference Fourier map and refined freely [N–H = 0.868 (19) Å]. The remaining H atoms were positioned geometrically and refined using a riding model with C–H = 0.93 or 0.97 Å and *U*_iso_(H) = 1.2 *U*_eq_(C).

### Crystal data

**Table:**

C_11_H_9_ClN_2_OS	*F*(000) = 520
*M**_r_* = 252.71	*D*_x_ = 1.463 Mg m^−^^3^
Monoclinic, *P*2_1_/*c*	Mo *K*α radiation, λ = 0.71073 Å
Hall symbol: -P 2ybc	Cell parameters from 4547 reflections
*a* = 13.9169 (13) Å	θ = 2.7–30.3°
*b* = 5.5188 (5) Å	µ = 0.49 mm^−^^1^
*c* = 15.1836 (14) Å	*T* = 296 K
β = 100.311 (2)°	Block, orange
*V* = 1147.34 (18) Å^3^	0.35 × 0.29 × 0.18 mm
*Z* = 4	

### Data collection

**Table:**

Bruker SMART APEXII DUO CCD diffractometer	3421 independent reflections
Radiation source: fine-focus sealed tube	2769 reflections with *I* > 2σ(*I*)
Graphite monochromator	*R*_int_ = 0.020
φ and ω scans	θ_max_ = 30.3°, θ_min_ = 1.5°
Absorption correction: multi-scan (*SADABS*; Bruker, 2009)	*h* = −19→19
*T*_min_ = 0.847, *T*_max_ = 0.918	*k* = −7→7
11463 measured reflections	*l* = −21→21

### Refinement

**Table:**

Refinement on *F*^2^	Primary atom site location: structure-invariant direct methods
Least-squares matrix: full	Secondary atom site location: difference Fourier map
*R*[*F*^2^ > 2σ(*F*^2^)] = 0.036	Hydrogen site location: inferred from neighbouring sites
*wR*(*F*^2^) = 0.116	H atoms treated by a mixture of independent and constrained refinement
*S* = 1.02	*w* = 1/[σ^2^(*F*_o_^2^) + (0.0629*P*)^2^ + 0.2113*P*] where *P* = (*F*_o_^2^ + 2*F*_c_^2^)/3
3421 reflections	(Δ/σ)_max_ = 0.001
149 parameters	Δρ_max_ = 0.29 e Å^−^^3^
0 restraints	Δρ_min_ = −0.29 e Å^−^^3^

### Special details

**Table:**

Geometry. All esds (except the esd in the dihedral angle between two l.s. planes) are estimated using the full covariance matrix. The cell esds are taken into account individually in the estimation of esds in distances, angles and torsion angles; correlations between esds in cell parameters are only used when they are defined by crystal symmetry. An approximate (isotropic) treatment of cell esds is used for estimating esds involving l.s. planes.
Refinement. Refinement of F^2^ against ALL reflections. The weighted R-factor wR and goodness of fit S are based on F^2^, conventional R-factors R are based on F, with F set to zero for negative F^2^. The threshold expression of F^2^ > 2sigma(F^2^) is used only for calculating R-factors(gt) etc. and is not relevant to the choice of reflections for refinement. R-factors based on F^2^ are statistically about twice as large as those based on F, and R- factors based on ALL data will be even larger.

### Fractional atomic coordinates and isotropic or equivalent isotropic displacement parameters (Å^2^)

**Table:**

	*x*	*y*	*z*	*U*_iso_*/*U*_eq_	
Cl1	−0.20822 (3)	0.54220 (12)	0.82496 (4)	0.08193 (19)	
S1	0.39710 (3)	1.03306 (7)	1.11917 (3)	0.04855 (13)	
O1	0.23806 (7)	0.8565 (2)	1.00294 (8)	0.0547 (3)	
N1	0.53067 (8)	0.7454 (2)	1.08411 (8)	0.0444 (3)	
N2	0.37558 (8)	0.6335 (2)	1.00962 (8)	0.0410 (2)	
C1	0.51611 (12)	1.0855 (3)	1.16882 (11)	0.0530 (4)	
H1A	0.5365	1.2115	1.2085	0.064*	
C2	0.57568 (11)	0.9199 (3)	1.14263 (11)	0.0503 (3)	
H2A	0.6429	0.9223	1.1626	0.060*	
C3	0.43688 (9)	0.7846 (2)	1.06618 (8)	0.0370 (3)	
C4	0.27847 (9)	0.6768 (2)	0.98005 (9)	0.0409 (3)	
C5	0.22953 (11)	0.4817 (3)	0.91743 (12)	0.0508 (4)	
H5A	0.2547	0.4902	0.8619	0.061*	
H5B	0.2473	0.3248	0.9442	0.061*	
C6	0.11993 (10)	0.4988 (2)	0.89603 (9)	0.0396 (3)	
C7	0.06207 (10)	0.3160 (2)	0.92138 (9)	0.0425 (3)	
H7A	0.0916	0.1835	0.9531	0.051*	
C8	−0.03890 (11)	0.3270 (3)	0.90033 (10)	0.0458 (3)	
H8A	−0.0773	0.2037	0.9174	0.055*	
C9	−0.08118 (10)	0.5259 (3)	0.85331 (9)	0.0457 (3)	
C10	−0.02621 (11)	0.7100 (3)	0.82739 (9)	0.0501 (3)	
H10A	−0.0562	0.8422	0.7958	0.060*	
C11	0.07454 (11)	0.6961 (3)	0.84901 (9)	0.0465 (3)	
H11A	0.1124	0.8204	0.8319	0.056*	
H1N2	0.4009 (14)	0.518 (3)	0.9827 (12)	0.052 (5)*	

### Atomic displacement parameters (Å^2^)

**Table:**

	*U*^11^	*U*^22^	*U*^33^	*U*^12^	*U*^13^	*U*^23^
Cl1	0.0398 (2)	0.1231 (5)	0.0797 (3)	0.0207 (2)	0.0020 (2)	0.0027 (3)
S1	0.0515 (2)	0.03917 (19)	0.0564 (2)	0.00832 (14)	0.01343 (16)	−0.00790 (14)
O1	0.0402 (5)	0.0473 (6)	0.0736 (7)	0.0117 (4)	0.0022 (5)	−0.0138 (5)
N1	0.0366 (5)	0.0441 (6)	0.0521 (6)	0.0018 (5)	0.0068 (5)	−0.0104 (5)
N2	0.0334 (5)	0.0361 (5)	0.0530 (6)	0.0044 (4)	0.0067 (5)	−0.0074 (5)
C1	0.0612 (9)	0.0455 (8)	0.0523 (8)	−0.0065 (7)	0.0099 (7)	−0.0129 (6)
C2	0.0433 (7)	0.0531 (8)	0.0529 (8)	−0.0051 (6)	0.0044 (6)	−0.0103 (6)
C3	0.0374 (6)	0.0328 (6)	0.0418 (6)	0.0036 (5)	0.0098 (5)	−0.0006 (5)
C4	0.0347 (6)	0.0398 (6)	0.0480 (7)	0.0047 (5)	0.0069 (5)	−0.0009 (5)
C5	0.0387 (7)	0.0504 (8)	0.0615 (9)	0.0064 (6)	0.0041 (6)	−0.0147 (7)
C6	0.0377 (6)	0.0405 (6)	0.0388 (6)	0.0062 (5)	0.0016 (5)	−0.0073 (5)
C7	0.0460 (7)	0.0368 (6)	0.0422 (6)	0.0087 (5)	0.0012 (5)	−0.0007 (5)
C8	0.0445 (7)	0.0466 (7)	0.0465 (7)	0.0006 (6)	0.0085 (6)	−0.0025 (6)
C9	0.0369 (6)	0.0582 (8)	0.0395 (6)	0.0115 (6)	0.0003 (5)	−0.0069 (6)
C10	0.0560 (8)	0.0472 (8)	0.0421 (7)	0.0150 (6)	−0.0051 (6)	0.0032 (6)
C11	0.0524 (8)	0.0415 (7)	0.0437 (7)	−0.0003 (6)	0.0033 (6)	0.0019 (5)

### Geometric parameters (Å, º)

**Table:**

Cl1—C9	1.7455 (15)	C5—C6	1.5045 (19)
S1—C1	1.7170 (17)	C5—H5A	0.9700
S1—C3	1.7300 (13)	C5—H5B	0.9700
O1—C4	1.2211 (16)	C6—C7	1.388 (2)
N1—C3	1.3029 (17)	C6—C11	1.3904 (19)
N1—C2	1.3820 (18)	C7—C8	1.386 (2)
N2—C4	1.3666 (16)	C7—H7A	0.9300
N2—C3	1.3778 (17)	C8—C9	1.382 (2)
N2—H1N2	0.868 (19)	C8—H8A	0.9300
C1—C2	1.341 (2)	C9—C10	1.371 (2)
C1—H1A	0.9300	C10—C11	1.384 (2)
C2—H2A	0.9300	C10—H10A	0.9300
C4—C5	1.515 (2)	C11—H11A	0.9300
			
C1—S1—C3	88.50 (7)	C4—C5—H5B	108.6
C3—N1—C2	109.91 (12)	H5A—C5—H5B	107.6
C4—N2—C3	124.38 (11)	C7—C6—C11	118.63 (13)
C4—N2—H1N2	115.8 (12)	C7—C6—C5	120.71 (13)
C3—N2—H1N2	118.8 (12)	C11—C6—C5	120.65 (14)
C2—C1—S1	110.73 (12)	C8—C7—C6	121.25 (13)
C2—C1—H1A	124.6	C8—C7—H7A	119.4
S1—C1—H1A	124.6	C6—C7—H7A	119.4
C1—C2—N1	115.61 (14)	C9—C8—C7	118.34 (14)
C1—C2—H2A	122.2	C9—C8—H8A	120.8
N1—C2—H2A	122.2	C7—C8—H8A	120.8
N1—C3—N2	121.01 (11)	C10—C9—C8	121.92 (13)
N1—C3—S1	115.24 (10)	C10—C9—Cl1	118.92 (11)
N2—C3—S1	123.75 (10)	C8—C9—Cl1	119.15 (13)
O1—C4—N2	121.81 (13)	C9—C10—C11	118.98 (13)
O1—C4—C5	125.28 (12)	C9—C10—H10A	120.5
N2—C4—C5	112.91 (11)	C11—C10—H10A	120.5
C6—C5—C4	114.56 (11)	C10—C11—C6	120.88 (14)
C6—C5—H5A	108.6	C10—C11—H11A	119.6
C4—C5—H5A	108.6	C6—C11—H11A	119.6
C6—C5—H5B	108.6		
			
C3—S1—C1—C2	0.48 (13)	C4—C5—C6—C7	−116.45 (15)
S1—C1—C2—N1	−0.8 (2)	C4—C5—C6—C11	64.72 (19)
C3—N1—C2—C1	0.7 (2)	C11—C6—C7—C8	0.3 (2)
C2—N1—C3—N2	−179.52 (13)	C5—C6—C7—C8	−178.55 (13)
C2—N1—C3—S1	−0.32 (16)	C6—C7—C8—C9	−0.1 (2)
C4—N2—C3—N1	−172.65 (13)	C7—C8—C9—C10	0.0 (2)
C4—N2—C3—S1	8.23 (19)	C7—C8—C9—Cl1	179.30 (11)
C1—S1—C3—N1	−0.08 (12)	C8—C9—C10—C11	0.0 (2)
C1—S1—C3—N2	179.09 (12)	Cl1—C9—C10—C11	−179.33 (11)
C3—N2—C4—O1	−0.6 (2)	C9—C10—C11—C6	0.2 (2)
C3—N2—C4—C5	179.13 (13)	C7—C6—C11—C10	−0.3 (2)
O1—C4—C5—C6	−10.4 (2)	C5—C6—C11—C10	178.52 (13)
N2—C4—C5—C6	169.89 (13)		

### Hydrogen-bond geometry (Å, º)

**Table:**

*D*—H···*A*	*D*—H	H···*A*	*D*···*A*	*D*—H···*A*
N2—H1*N*2···N1^i^	0.866 (18)	2.096 (18)	2.9606 (16)	176.2 (17)

Symmetry code: (i) −*x*+1, −*y*+1, −*z*+2.
